# (2*R*,3*R*,4a*S*,6*S*,7*S*,8a*S*)-4a-Fluoro-8a-hy­droxy­perhydro­naphthalene-2,3,6,7-tetrayl tetraacetate

**DOI:** 10.1107/S1600536810044314

**Published:** 2010-11-06

**Authors:** Goverdhan Mehta, Saikat Sen

**Affiliations:** a Department of Organic Chemistry, Indian Institute of Science, Bangalore 560 012, Karnataka, India

## Abstract

The title compound, C_18_H_25_FO_9_, exhibits a similar unit cell and packing to the α polymorph of axial 4a,8a-dihy­droxy­per­hydro­naph­tha­lene-2,3,6,7-tetrayl tetraacetate. The carbonyl O atoms of two of the four acetate groups in the molecule are disordered over two sites with occupancy ratios of 0.59 (4):0.41 (4) and 0.57 (6):0.43 (6). Crystal packing is effected *via* inter­molecular O—H⋯O hydrogen bonds, which link the tetra­acetate mol­ecules into tapes along the *c* axis.

## Related literature

The synthesis and spectral characterization of the title compound have already been communicated (Mehta & Sen, 2010*c* ▶). For the α polymorph of tetraacetate, see: Mehta & Sen (2009*a* ▶,*b* ▶, 2010*a* ▶,*b* ▶). For determination of absolute structure, see: Flack (1983 ▶); Flack & Bernardinelli (2000 ▶).
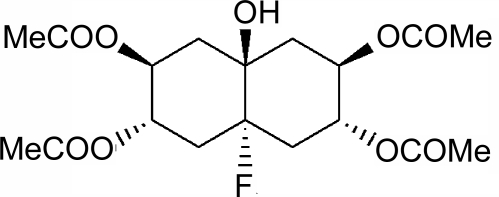


## Experimental

### 

#### Crystal data

C_18_H_25_FO_9_
*M* *_r_* = 404.38
Monoclinic, 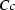

*a* = 21.144 (3) Å
*b* = 5.6497 (7) Å
*c* = 16.898 (2) Å
β = 104.290 (6)°
*V* = 1956.2 (4) Å^3^
*Z* = 4
Mo *K*α radiation
μ = 0.12 mm^−1^
*T* = 291 K
0.27 × 0.23 × 0.03 mm

#### Data collection

Bruker SMART APEX CCD area-detector diffractometer
Absorption correction: multi-scan (*SADABS*; Sheldrick, 2003 ▶) *T*_min_ = 0.969, *T*_max_ = 0.997
12391 measured reflections
1980 independent reflections
1290 reflections with *I* > 2σ(*I*)
*R*_int_ = 0.042

#### Refinement

*R*[*F* ^2^ > 2σ(*F* ^2^)] = 0.043
*wR*(*F* ^2^) = 0.167
*S* = 1.13
1980 reflections
278 parameters
2 restraints
H-atom parameters constrained
Δρ_max_ = 0.32 e Å^−3^
Δρ_min_ = −0.40 e Å^−3^

### 

Data collection: *SMART* (Bruker, 1998 ▶); cell refinement: *SMART*; data reduction: *SAINT* (Bruker, 1998 ▶); program(s) used to solve structure: *SIR92* (Altomare *et al.*, 1994 ▶); program(s) used to refine structure: *SHELXL97* (Sheldrick, 2008 ▶); molecular graphics: *ORTEP-3 for Windows* (Farrugia, 1997 ▶) and *CAMERON* (Watkin *et al.*, 1993 ▶); software used to prepare material for publication: *PLATON* (Spek, 2009 ▶).

## Supplementary Material

Crystal structure: contains datablocks global, I. DOI: 10.1107/S1600536810044314/pb2046sup1.cif

Structure factors: contains datablocks I. DOI: 10.1107/S1600536810044314/pb2046Isup2.hkl

Additional supplementary materials:  crystallographic information; 3D view; checkCIF report

## Figures and Tables

**Table 1:** Hydrogen-bond geometry (Å, °)

*D*—H⋯*A*	*D*—H	H⋯*A*	*D*⋯*A*	*D*—H⋯*A*
O3—H3⋯O6^i^	0.82	2.47	3.174 (6)	144

Symmetry code: (i) 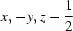.

**Fig. 1.**
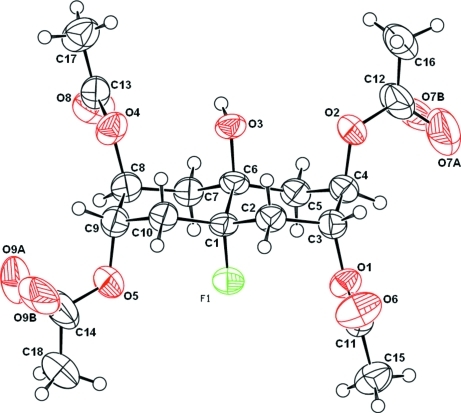
View of the tetra-acetate 1, with the atom numbering scheme. Displacement ellipsoids for non-H atoms are drawn at the 50% probability level. H atoms are shown as small spheres of arbitrary radii.

**Fig. 2.**
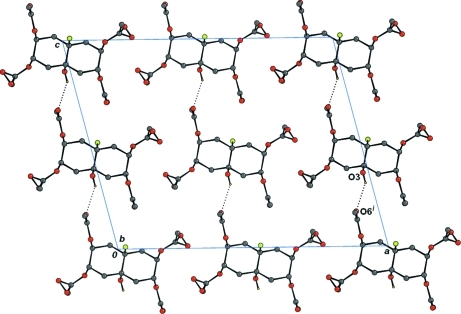
The molecular packing of 1. H atoms not involved in H-bonding have been omitted for clarity. Dotted lines indicate hydrogen bonds.

**Fig. 3.**
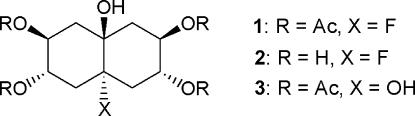
The structure of (1), (2) and (3).

## supplementary crystallographic information

### Comment

The title compound 1 is the tetra-acetate derivative of the monofluoropentol 2 whose synthesis and crystal structure elucidation have been reported by us recently (Mehta & Sen, 2010*a*). The C_s_ symmetric molecule 1 crystallized in the non-centrosymmetric space group *Cc* (*Z* = 4) and was found to display an interesting iso-structurality with the α polymorph of the tetra-acetate 3 (Mehta & Sen, 2009*a*, 2009*b* and 2010*b*). It is pertinent to mention that the tetra-acetates 3 and 1 are isosteric with a fluoro group in 1 replacing a hydroxy substituent in 3. The crystal structure of α polymorphic modification of 3 had been solved in the centrosymmetric monoclinic space group *C*2/*c* (*a* = 21.433 (7), *b* = 5.7126 (18), *c* = 16.720 (5) Å, β = 105.664 (5)°, *V* = 1971.1 (11) Å^3^, *Z* = 4, *T* = 291 K), and the C_2 h_ symmetric tetra-acetate molecules were found to occupy the inversion centers at (1/2, 0, 1/2), (1/2, 0, 0), (0, 1/2, 0) and (0, 1/2, 1/2).

Quite akin to that observed in the α form of 3, the carbonyl O atoms (O7 and O9) of two acetate groups in the asymmetric unit of 1 are disordered over two sites, A and B, having occupancy factors of about 0.60 and 0.40 respectively (Fig. 1). The tertiary hydroxyl group in 1 does not engage itself as an intramolecular O—H···O hydrogen bond donor to either of the flanking 1,3-syndiaxial oxygen acceptors, O2 and O4.

Similar again to the favored mode of self-assembly in 3 (Mehta & Sen, 2009*a*, 2009*b* and 2010*b*), molecular packing in 1 is effected *via* the agency of intermolecular O—H···O hydrogen bonds which link the tetra-acetate molecules into chains along the *c* axis (Fig. 2). A soft intermolecular C—H···F contact (C17—H17A···F1, d = 2.44 Å, θ = 154°) exists between successive molecules in the H-bonded chains thus formed. Intermolecular C—H···O contacts (C16—H16A···O8, d = 2.59 Å, θ = 174°) can also be discerned between the translationally related molecular chains.

### Experimental

The title compound was prepared by acetylating the monofluoropentol 2 at ambient temperature in presence of acetic anhydride and 4-Dimethylaminopyridine (Mehta & Sen, 2010*c*). Single crystals of 1, suitable for X-ray diffraction studies, were grown by slow solvent evaporation of its solution in 1:1 dichloromethane and petroleum ether under ambient temperature and pressure.

### Refinement

The methine (CH) and methylene (CH_2_) H atoms were placed in geometrically idealized positions and allowed to ride on their parent atoms with C—H distances in the range 0.97–0.98 Å and *U*_iso_(H) = 1.2*U*_eq_(C). The CH_3_ and OH hydrogen atoms were constrained to an ideal geometry with C—H distances as 0.96 Å and *U*_iso_(H) = 1.5*U*_eq_(C), and O—H distances fixed at 0.82 Å and *U*_iso_(H) = 1.5*U*_eq_(O). During refinement, each methyl and hydroxyl group was however allowed to rotate freely about its C—C and C—O bond respectively. Due to the absence of any significant anomalous scatterers (*Z*>Si) in 1, attempts to refine the Flack (Flack, 1983) parameter led to an inconclusive value of -0.8 (13) (Flack & Bernardinelli, 2000). Therefore the intensities of the Friedel pairs (1828) were averaged prior to merging of data in *Cc*, so that the reported value of *R*_int_ corresponds to subsequent merging of equivalent reflections in this space group.

### Crystal data

**Table:**

C_18_H_25_FO_9_	*F*(000) = 856
*M**_r_* = 404.38	*D*_x_ = 1.373 Mg m^−^^3^
Monoclinic, *C**c*	Mo *K*α radiation, λ = 0.71073 Å
Hall symbol: C -2yc	Cell parameters from 2973 reflections
*a* = 21.144 (3) Å	θ = 2.5–22.3°
*b* = 5.6497 (7) Å	µ = 0.12 mm^−^^1^
*c* = 16.898 (2) Å	*T* = 291 K
β = 104.290 (6)°	Plate, colorless
*V* = 1956.2 (4) Å^3^	0.27 × 0.23 × 0.03 mm
*Z* = 4	

### Data collection

**Table:**

Bruker SMART APEX CCD area-detector diffractometer	1980 independent reflections
Radiation source: fine-focus sealed tube	1290 reflections with *I* > 2σ(*I*)
graphite	*R*_int_ = 0.042
φ and ω scans	θ_max_ = 26.4°, θ_min_ = 2.0°
Absorption correction: multi-scan (*SADABS*; Sheldrick, 2003)	*h* = −26→26
*T*_min_ = 0.969, *T*_max_ = 0.997	*k* = −6→7
12391 measured reflections	*l* = −21→21

### Refinement

**Table:**

Refinement on *F*^2^	Primary atom site location: structure-invariant direct methods
Least-squares matrix: full	Secondary atom site location: difference Fourier map
*R*[*F*^2^ > 2σ(*F*^2^)] = 0.043	Hydrogen site location: inferred from neighbouring sites
*wR*(*F*^2^) = 0.167	H-atom parameters constrained
*S* = 1.13	*w* = 1/[σ^2^(*F*_o_^2^) + (0.0992*P*)^2^] where *P* = (*F*_o_^2^ + 2*F*_c_^2^)/3
1980 reflections	(Δ/σ)_max_ = 0.001
278 parameters	Δρ_max_ = 0.32 e Å^−^^3^
2 restraints	Δρ_min_ = −0.40 e Å^−^^3^

### Special details

**Table:**

Geometry. All e.s.d.'s (except the e.s.d. in the dihedral angle between two l.s. planes) are estimated using the full covariance matrix. The cell e.s.d.'s are taken into account individually in the estimation of e.s.d.'s in distances, angles and torsion angles; correlations between e.s.d.'s in cell parameters are only used when they are defined by crystal symmetry. An approximate (isotropic) treatment of cell e.s.d.'s is used for estimating e.s.d.'s involving l.s. planes.
Refinement. Refinement of *F*^2^ against ALL reflections. The weighted *R*-factor *wR* and goodness of fit *S* are based on *F*^2^, conventional *R*-factors *R* are based on *F*, with *F* set to zero for negative *F*^2^. The threshold expression of *F*^2^ > σ(*F*^2^) is used only for calculating *R*-factors(gt) *etc*. and is not relevant to the choice of reflections for refinement. *R*-factors based on *F*^2^ are statistically about twice as large as those based on *F*, and *R*- factors based on ALL data will be even larger.

### Fractional atomic coordinates and isotropic or equivalent isotropic displacement parameters (Å^2^)

**Table:**

	*x*	*y*	*z*	*U*_iso_*/*U*_eq_	Occ. (<1)
F1	1.02959 (14)	0.2496 (5)	0.50960 (17)	0.0507 (8)	
O1	0.90847 (16)	0.2234 (6)	0.5479 (2)	0.0488 (9)	
O2	0.85104 (18)	−0.1582 (7)	0.3760 (2)	0.0585 (10)	
O3	0.97848 (17)	−0.1802 (6)	0.3515 (2)	0.0461 (8)	
O4	1.10283 (18)	−0.1377 (7)	0.3161 (2)	0.0572 (10)	
O5	1.15663 (18)	0.2400 (8)	0.4902 (3)	0.0646 (11)	
O6	0.9155 (3)	0.0418 (8)	0.6668 (3)	0.0776 (15)	
O7A	0.7494 (6)	−0.026 (3)	0.353 (3)	0.141 (12)	0.59 (4)
O7B	0.7688 (13)	0.020 (2)	0.2957 (17)	0.084 (11)	0.41 (4)
O8	1.1025 (2)	0.0561 (9)	0.2014 (2)	0.0763 (13)	
O9A	1.2601 (6)	0.111 (4)	0.511 (3)	0.132 (11)	0.57 (6)
O9B	1.2443 (18)	0.069 (3)	0.561 (2)	0.100 (14)	0.43 (6)
C1	1.0204 (2)	0.0190 (9)	0.4762 (3)	0.0405 (11)	
C2	0.9740 (3)	−0.1125 (10)	0.5186 (3)	0.0462 (13)	
C3	0.9065 (2)	−0.0036 (10)	0.5062 (3)	0.0473 (12)	
C4	0.8755 (2)	0.0617 (10)	0.4160 (3)	0.0483 (13)	
C5	0.9226 (2)	0.1789 (10)	0.3728 (3)	0.0473 (12)	
C6	0.9888 (2)	0.0525 (9)	0.3845 (3)	0.0418 (12)	
C7	1.0343 (2)	0.1924 (10)	0.3440 (3)	0.0469 (13)	
C8	1.1028 (3)	0.0865 (11)	0.3587 (3)	0.0530 (13)	
C9	1.1335 (3)	0.0193 (10)	0.4480 (3)	0.0530 (13)	
C10	1.0862 (2)	−0.1011 (10)	0.4896 (3)	0.0479 (13)	
C11	0.9114 (3)	0.2201 (11)	0.6278 (3)	0.0510 (14)	
C12	0.7910 (3)	−0.1666 (15)	0.3313 (6)	0.084 (2)	
C13	1.1032 (3)	−0.1261 (12)	0.2372 (3)	0.0545 (15)	
C14	1.2182 (4)	0.2519 (15)	0.5306 (6)	0.090 (3)	
C15	0.9102 (3)	0.4608 (12)	0.6605 (4)	0.0702 (18)	
C16	0.7719 (3)	−0.3971 (14)	0.2935 (5)	0.087 (2)	
C17	1.1045 (4)	−0.3680 (13)	0.2028 (4)	0.0739 (19)	
C18	1.2354 (4)	0.4829 (16)	0.5723 (5)	0.090 (2)	
H2A	0.9691	−0.2741	0.4986	0.055*	
H2B	0.9941	−0.1187	0.5767	0.055*	
H3	0.9780	−0.1766	0.3028	0.069*	
H3A	0.8778	−0.1130	0.5258	0.057*	
H4	0.8387	0.1686	0.4138	0.058*	
H5A	0.9303	0.3401	0.3925	0.057*	
H5B	0.9019	0.1864	0.3148	0.057*	
H7A	1.0151	0.1993	0.2857	0.056*	
H7B	1.0378	0.3533	0.3647	0.056*	
H8	1.1314	0.1988	0.3402	0.064*	
H9	1.1708	−0.0855	0.4502	0.064*	
H10A	1.0794	−0.2626	0.4698	0.057*	
H10B	1.1059	−0.1080	0.5478	0.057*	
H15A	0.9182	0.4538	0.7189	0.105*	
H15B	0.8682	0.5307	0.6381	0.105*	
H15C	0.9433	0.5549	0.6458	0.105*	
H16A	0.7260	−0.3970	0.2682	0.131*	
H16B	0.7816	−0.5184	0.3345	0.131*	
H16C	0.7957	−0.4272	0.2529	0.131*	
H17A	1.0957	−0.3586	0.1444	0.111*	
H17B	1.0719	−0.4643	0.2178	0.111*	
H17C	1.1468	−0.4371	0.2240	0.111*	
H18A	1.1969	0.5542	0.5823	0.136*	
H18B	1.2535	0.5855	0.5382	0.136*	
H18C	1.2669	0.4585	0.6233	0.136*	

### Atomic displacement parameters (Å^2^)

**Table:**

	*U*^11^	*U*^22^	*U*^33^	*U*^12^	*U*^13^	*U*^23^
F1	0.0532 (17)	0.060 (2)	0.0363 (16)	−0.0023 (14)	0.0062 (13)	−0.0073 (13)
O1	0.059 (2)	0.056 (2)	0.033 (2)	0.0057 (16)	0.0142 (15)	0.0009 (15)
O2	0.040 (2)	0.074 (3)	0.055 (2)	−0.0031 (17)	−0.0005 (18)	−0.0122 (19)
O3	0.053 (2)	0.054 (2)	0.0303 (18)	0.0020 (16)	0.0073 (16)	−0.0082 (15)
O4	0.061 (2)	0.076 (3)	0.038 (2)	0.0094 (19)	0.0192 (18)	−0.0002 (18)
O5	0.043 (2)	0.084 (3)	0.059 (3)	−0.0022 (18)	−0.0003 (19)	−0.012 (2)
O6	0.127 (4)	0.068 (3)	0.043 (3)	0.014 (3)	0.029 (3)	0.011 (2)
O7A	0.054 (6)	0.126 (10)	0.23 (3)	0.008 (6)	0.008 (10)	−0.049 (14)
O7B	0.060 (13)	0.062 (9)	0.099 (16)	0.008 (6)	−0.036 (9)	−0.011 (7)
O8	0.097 (3)	0.094 (4)	0.038 (2)	−0.013 (3)	0.018 (2)	0.002 (2)
O9A	0.049 (6)	0.150 (13)	0.19 (3)	0.007 (6)	0.026 (9)	−0.045 (15)
O9B	0.062 (16)	0.100 (12)	0.104 (19)	0.020 (8)	−0.045 (12)	−0.017 (10)
C1	0.046 (3)	0.048 (3)	0.026 (2)	−0.001 (2)	0.006 (2)	−0.006 (2)
C2	0.052 (3)	0.053 (3)	0.032 (3)	0.003 (2)	0.006 (2)	0.001 (2)
C3	0.044 (3)	0.057 (3)	0.040 (3)	−0.001 (2)	0.009 (2)	−0.001 (2)
C4	0.044 (3)	0.057 (4)	0.039 (3)	0.002 (2)	0.000 (2)	−0.003 (2)
C5	0.047 (3)	0.057 (3)	0.032 (3)	0.005 (2)	0.000 (2)	0.000 (2)
C6	0.046 (3)	0.049 (3)	0.026 (2)	−0.002 (2)	0.001 (2)	−0.0068 (19)
C7	0.049 (3)	0.062 (4)	0.029 (3)	0.003 (2)	0.007 (2)	0.002 (2)
C8	0.053 (3)	0.073 (4)	0.033 (3)	0.000 (3)	0.011 (2)	−0.002 (2)
C9	0.044 (3)	0.073 (4)	0.040 (3)	0.006 (2)	0.007 (2)	−0.003 (3)
C10	0.046 (3)	0.068 (4)	0.027 (3)	0.010 (2)	0.004 (2)	0.001 (2)
C11	0.046 (3)	0.071 (4)	0.035 (3)	0.008 (2)	0.008 (2)	−0.002 (3)
C12	0.049 (4)	0.089 (6)	0.096 (6)	0.005 (3)	−0.017 (4)	−0.016 (4)
C13	0.046 (3)	0.082 (5)	0.034 (3)	0.006 (3)	0.008 (2)	0.001 (3)
C14	0.048 (5)	0.092 (6)	0.114 (7)	−0.007 (4)	−0.012 (4)	−0.009 (5)
C15	0.085 (5)	0.074 (5)	0.052 (4)	0.012 (3)	0.018 (3)	−0.008 (3)
C16	0.052 (4)	0.088 (5)	0.111 (6)	−0.011 (3)	−0.003 (4)	−0.023 (4)
C17	0.082 (5)	0.093 (5)	0.046 (4)	0.018 (4)	0.013 (3)	−0.007 (3)
C18	0.061 (4)	0.109 (6)	0.088 (6)	−0.016 (4)	−0.005 (4)	−0.019 (4)

### Geometric parameters (Å, °)

**Table:**

F1—C1	1.414 (6)	C6—C5	1.540 (7)
O1—C3	1.459 (7)	C6—C7	1.531 (7)
O1—C11	1.335 (6)	C7—C8	1.530 (8)
O2—C12	1.309 (7)	C7—H7A	0.9700
O2—C4	1.447 (6)	C7—H7B	0.9700
O3—C6	1.424 (6)	C8—H8	0.9800
O3—H3	0.8200	C9—C8	1.536 (8)
O4—C8	1.457 (7)	C9—H9	0.9800
O4—C13	1.337 (7)	C10—C1	1.514 (7)
O5—C9	1.460 (7)	C10—C9	1.519 (8)
O5—C14	1.314 (8)	C10—H10A	0.9700
O6—C11	1.195 (7)	C10—H10B	0.9700
O7A—C12	1.30 (2)	C11—C15	1.470 (9)
O7B—C12	1.246 (16)	C12—C16	1.463 (10)
O8—C13	1.192 (8)	C13—C17	1.488 (9)
O9A—C14	1.294 (18)	C15—H15A	0.9600
O9B—C14	1.226 (17)	C15—H15B	0.9600
C2—C1	1.541 (7)	C15—H15C	0.9600
C2—C3	1.520 (7)	C16—H16A	0.9600
C2—H2A	0.9700	C16—H16B	0.9600
C2—H2B	0.9700	C16—H16C	0.9600
C3—H3A	0.9800	C17—H17A	0.9600
C4—C3	1.548 (7)	C17—H17B	0.9600
C4—H4	0.9800	C17—H17C	0.9600
C5—C4	1.522 (7)	C18—C14	1.485 (12)
C5—H5A	0.9700	C18—H18A	0.9600
C5—H5B	0.9700	C18—H18B	0.9600
C6—C1	1.540 (5)	C18—H18C	0.9600
			
F1—C1—C2	107.5 (4)	C6—O3—H3	109.5
F1—C1—C6	105.7 (4)	C6—C1—C2	111.0 (4)
F1—C1—C10	108.8 (4)	C6—C5—H5A	108.6
O1—C3—C2	112.0 (4)	C6—C5—H5B	108.6
O1—C3—C4	102.8 (4)	C6—C7—H7A	108.8
O1—C3—H3A	109.7	C6—C7—H7B	108.8
O1—C11—C15	111.5 (5)	C7—C6—C1	110.4 (4)
O2—C4—C3	105.7 (4)	C7—C6—C5	110.8 (4)
O2—C4—C5	110.5 (4)	C7—C8—C9	114.2 (4)
O2—C4—H4	108.8	C7—C8—H8	109.2
O2—C12—C16	114.1 (6)	C8—C7—C6	113.6 (4)
O3—C6—C1	105.5 (4)	C8—C7—H7A	108.8
O3—C6—C5	109.3 (4)	C8—C7—H7B	108.8
O3—C6—C7	110.7 (4)	C8—C9—H9	109.2
O4—C8—C7	112.1 (4)	C9—C8—H8	109.2
O4—C8—C9	102.7 (4)	C9—C10—H10A	108.6
O4—C8—H8	109.2	C9—C10—H10B	108.6
O4—C13—C17	110.5 (6)	C10—C1—C2	112.4 (4)
O5—C9—C8	106.2 (5)	C10—C1—C6	111.1 (3)
O5—C9—C10	109.6 (4)	C10—C9—C8	113.4 (4)
O5—C14—C18	112.7 (7)	C10—C9—H9	109.2
O5—C9—H9	109.2	C11—O1—C3	117.7 (4)
O6—C11—O1	123.2 (6)	C11—C15—H15A	109.5
O6—C11—C15	125.3 (6)	C11—C15—H15B	109.5
O7A—C12—O2	116.2 (12)	C11—C15—H15C	109.5
O7A—C12—C16	122.3 (9)	C12—O2—C4	119.0 (5)
O7B—C12—O2	116.4 (11)	C12—C16—H16A	109.5
O7B—C12—O7B	54.6 (9)	C12—C16—H16B	109.5
O7B—C12—C16	120.7 (9)	C12—C16—H16C	109.5
O8—C13—O4	123.1 (6)	C13—O4—C8	116.8 (5)
O8—C13—C17	126.5 (6)	C13—C17—H17A	109.5
O9A—C14—O5	119.2 (14)	C13—C17—H17B	109.5
O9A—C14—C18	124.1 (9)	C13—C17—H17C	109.5
O9B—C14—O5	117.5 (15)	C14—O5—C9	117.8 (5)
O9B—C14—O9A	47.4 (10)	C14—C18—H18A	109.5
O9B—C14—C18	120.6 (11)	C14—C18—H18B	109.5
C1—C2—H2A	108.5	C14—C18—H18C	109.5
C1—C2—H2B	108.5	H2A—C2—H2B	107.5
C1—C10—C9	114.6 (5)	H5A—C5—H5B	107.6
C1—C10—H10A	108.6	H7A—C7—H7B	107.7
C1—C10—H10B	108.6	H10A—C10—H10B	107.6
C2—C3—C4	112.8 (4)	H15A—C15—H15B	109.5
C2—C3—H3A	109.7	H15A—C15—H15C	109.5
C3—C2—C1	115.1 (4)	H15B—C15—H15C	109.5
C3—C2—H2A	108.5	H16A—C16—H16B	109.5
C3—C2—H2B	108.5	H16A—C16—H16C	109.5
C3—C4—H4	108.8	H16B—C16—H16C	109.5
C4—C3—H3A	109.7	H17A—C17—H17B	109.5
C4—C5—C6	114.6 (4)	H17A—C17—H17C	109.5
C4—C5—H5A	108.6	H17B—C17—H17C	109.5
C4—C5—H5B	108.6	H18A—C18—H18B	109.5
C5—C4—C3	114.1 (4)	H18A—C18—H18C	109.5
C5—C4—H4	108.8	H18B—C18—H18C	109.5
C5—C6—C1	110.0 (3)		
			
O2—C4—C3—O1	−160.4 (4)	C5—C6—C7—C8	175.8 (4)
O2—C4—C3—C2	78.8 (5)	C6—C7—C8—O4	69.3 (5)
O3—C6—C1—F1	−179.0 (4)	C6—C5—C4—C3	47.7 (6)
O3—C6—C1—C2	−62.7 (4)	C6—C5—C4—O2	−71.2 (5)
O3—C6—C1—C10	63.2 (5)	C6—C7—C8—C9	−47.0 (6)
O3—C6—C5—C4	61.7 (5)	C7—C6—C1—F1	61.4 (4)
O3—C6—C7—C8	−62.8 (5)	C7—C6—C1—C2	177.7 (5)
O5—C9—C8—O4	160.3 (4)	C7—C6—C5—C4	−176.0 (4)
O5—C9—C8—C7	−78.1 (5)	C7—C6—C1—C10	−56.4 (5)
C1—C2—C3—O1	−68.8 (5)	C8—O4—C13—O8	−1.1 (8)
C1—C2—C3—C4	46.6 (6)	C8—O4—C13—C17	178.9 (5)
C1—C6—C5—C4	−53.7 (5)	C9—C10—C1—F1	−62.0 (5)
C1—C6—C7—C8	53.7 (5)	C9—C10—C1—C2	179.1 (4)
C1—C10—C9—C8	−46.6 (6)	C9—C10—C1—C6	54.0 (6)
C1—C10—C9—O5	71.9 (5)	C9—O5—C14—O9A	23 (3)
C3—O1—C11—O6	3.5 (8)	C9—O5—C14—O9B	−32 (3)
C3—O1—C11—C15	−177.5 (4)	C9—O5—C14—C18	−178.8 (6)
C3—C2—C1—F1	61.5 (5)	C10—C9—C8—O4	−79.2 (5)
C3—C2—C1—C6	−53.7 (5)	C10—C9—C8—C7	42.3 (7)
C3—C2—C1—C10	−178.8 (4)	C11—O1—C3—C2	−81.0 (5)
C4—O2—C12—O7A	−30 (2)	C11—O1—C3—C4	157.6 (4)
C4—O2—C12—O7B	32 (2)	C12—O2—C4—C3	130.1 (6)
C4—O2—C12—C16	179.5 (6)	C12—O2—C4—C5	−105.9 (7)
C5—C4—C3—O1	78.0 (5)	C13—O4—C8—C7	82.2 (6)
C5—C4—C3—C2	−42.8 (6)	C13—O4—C8—C9	−154.8 (4)
C5—C6—C1—F1	−61.2 (5)	C14—O5—C9—C8	−125.4 (7)
C5—C6—C1—C10	−179.0 (5)	C14—O5—C9—C10	111.7 (7)
C5—C6—C1—C2	55.1 (5)		

### Hydrogen-bond geometry (Å, °)

**Table:**

*D*—H···*A*	*D*—H	H···*A*	*D*···*A*	*D*—H···*A*
O3—H3···O6^i^	0.82	2.47	3.174 (6)	144

Symmetry codes: (i) *x*, −*y*, *z*−1/2.
